# Genetic Correlates of Individual Differences in Sleep Behavior of Free-Living Great Tits (*Parus major*)

**DOI:** 10.1534/g3.115.024216

**Published:** 2016-01-05

**Authors:** Erica F. Stuber, Christine Baumgartner, Niels J. Dingemanse, Bart Kempenaers, Jakob C. Mueller

**Affiliations:** * Department of Behavioural Ecology and Evolutionary Genetics, Max Planck Institute for Ornithology, Seewiesen, Germany; † Evolutionary Ecology of Variation Research Group, Max Planck Institute for Ornithology, 82319 Seewiesen, Germany; ‡ Department of Biology II, Behavioural Ecology, Ludwig–Maximilians University of Munich, 82152 Germany

**Keywords:** candidate gene, circadian rhythm, genotype-phenotype association, repeatability, sleep

## Abstract

Within populations, free-living birds display considerable variation in observable sleep behaviors, reflecting dynamic interactions between individuals and their environment. Genes are expected to contribute to repeatable between-individual differences in sleep behaviors, which may be associated with individual fitness. We identified and genotyped polymorphisms in nine candidate genes for sleep, and measured five repeatable sleep behaviors in free-living great tits (*Parus major*), partly replicating a previous study in blue tits (*Cyanistes caeruleus*). Microsatellites in the CLOCK and NPAS2 clock genes exhibited an association with sleep duration relative to night length, and morning latency to exit the nest box, respectively. Furthermore, microsatellites in the NPSR1 and PCSK2 genes associated with relative sleep duration and proportion of time spent awake at night, respectively. Given the detection rate of associations in the same models run with random markers instead of candidate genes, we expected two associations to arise by chance. The detection of four associations between candidate genes and sleep, however, suggests that clock genes, a clock-related gene, or a gene involved in the melanocortin system, could play key roles in maintaining phenotypic variation in sleep behavior in avian populations. Knowledge of the genetic architecture underlying sleep behavior in the wild is important because it will enable ecologists to assess the evolution of sleep in response to selection.

There is general interest in how phenotypic variation is maintained within and between populations and species (Dall *et al.* 2004; Moran 1992; Hallgrímsson and Hall 2011); studying genetic variation between individuals can provide mechanistic and evolutionary insight into the underpinnings of repeatable differences in behaviors that are heritable (van Oers *et al.* 2005; Mousseau *et al.* 2000; Boake 1989). Here, we refer to repeatability as individual consistency in behaviors over time (for a formal definition, see Nakagawa and Schielzeth 2010). Many behavioral traits show low-to-moderate heritability (Stirling *et al.* 2002), and many studies have quantified the genetic basis of individual variation in behavior, utilizing quantitative genetics to assess the extent to which repeatable variation in behavior is due to additive genetic effects (Réale *et al.* 2007; Dochtermann and Dingemanse 2013; Bakker 1999). However, studies regarding the specific genetic basis of overt behavioral phenotypes in ecological contexts are still scarce. Knowledge of the genetic architecture underlying variation in quantitative traits will enable us to fully understand the mechanisms of behavior.

The candidate gene approach enables behavioral ecologists to study the relationships between genotype and phenotype in nongenetic model organisms by borrowing information from genetic studies of classic model organisms to identify genes potentially involved in ecologically relevant behaviors (Fitzpatrick *et al.* 2005). Previous candidate gene studies have revealed that polymorphisms in certain genes are conserved across different species, and regulate similar behavioral phenotypes (van Oers *et al.* 2005; Toth and Robinson 2007; Lakin-Thomas 2000). Exploring the dynamics of candidate genes in naturally occurring populations opens avenues for addressing fundamental questions in ecology and evolution, including whether behavioral traits are influenced by few genes with large effects, how selection influences the distribution of genetic diversity, how genes may interact with the environment to influence plasticity and fitness, and whether common genes may underlie behavioral phenotypes in different species (Fitzpatrick *et al.* 2005; van Oers and Mueller 2010).

Sleep behavior is recognized as an ecologically relevant behavior for individuals as it has implications for energy balance (Laposky *et al.* 2008; Zepelin and Rechtschaffen 1974), and fitness via its effects on physical and cognitive performance (Koslowsky and Babkoff 1992; Lesku *et al.* 2012). Sleep behaviors are moderately heritable (Ambrosius *et al.* 2008; Gottlieb *et al.* 2007; Partinen *et al.* 1983), and individuals show repeatable differences in observable sleep components (Stuber *et al.* 2015; Steinmeyer *et al.* 2010), suggesting that these repeatable behaviors might be regulated by underlying genetic mechanisms. Genome-wide association studies (GWAS) performed in humans and other mammals have been successful in highlighting candidate genes for various behavioral and physiological sleep traits (see *Materials and Methods* section *Identifying candidate genes*). The great tit (*Parus major*) is a model organism for ecological research, and its behavior is well studied. Furthermore, the great tit is one of few species for which the variation in sleep behavior has been characterized under natural contexts (Stuber *et al.* 2015). We have identified five sleep behaviors that are repeatable between individuals in the wild, and thus may have a genetic basis (see *Materials and Methods* section *Behavioral sleep data*) (Stuber *et al.* 2015). However, we are aware of only one genotype-phenotype association study of sleep in birds, namely blue tits (Steinmeyer *et al.* 2012). The authors of this study demonstrate associations between four single nucleotide polymorphisms (SNPs) in clock genes and awakening time, morning latency, or the duration of the longest sleep bout. However, these associations did not satisfy study-wide significance.

In the present study, we aim to test the generalizability of the potential associations between sleep phenotypes and candidate genes identified in previous work in blue tits and primarily in mammals under highly controlled experimental conditions. Specifically, we aim to test whether an association exists between putative sleep genes and repeatable behavioral sleep traits in free-living great tits under natural conditions.

## Materials and Methods

### Study population

Data for this study were collected from a population of wild great tits roosting in nest boxes in 12 plots established in 2009 in Bavaria, Germany, southwest of Munich (47°58′ N, 11°14′ E). Each plot consists of a 9- to 12-ha forested area with 50 nest boxes. Each winter we captured, marked, and collected blood samples (which were subsequently used for genotyping: see Supporting Information, File S1 for details regarding DNA sampling, extraction, and genotyping) from all birds roosting in the nest boxes (see Stuber *et al.* 2015 for details). Sleep behaviors were recorded during December, February, and March of the winter seasons 2011/2012 and 2012/2013. In total, we obtained 246 recordings of 127 individual great tits during the two winter seasons.

### Behavioral sleep data

Behavioral sleep data were quantified from video recordings made on previously identified individuals; for a detailed description of field procedures for sleep recording, see Stuber *et al.* (2015). Briefly, one night prior to sleep recording, we performed night checks of each study site in semi-random order to locate great tits roosting in nest boxes. The following day, we installed infra-red video cameras (Conrad Electronic, www.Conrad.de) in each nest box where a great tit was previously found sleeping (between 2 hr after sunrise, and 2 hr before sunset, when nest boxes are unoccupied). We programmed the video cameras to record from 1 hr before sunset to 1 hr after sunrise to capture individuals’ entire sleep cycle. In this study, we defined sleep entirely by behavior. Birds were considered asleep when they adopted the classical sleep posture (Amlaner and Ball 1983), and considered awake when the beak and head were forward-facing or otherwise actively moving. Previous work in other bird species demonstrated a close correspondence between physiological and behavioral measures of sleep, lending credibility to strictly behavioral studies of sleep (Jones *et al.* 2008; Costa 2009; Lesku *et al.* 2011; Szymczak *et al.* 1993). Nevertheless, it remains possible that specific electrophysiological measures of sleep deviate from behavioral patterns, and thus would present different relationships with candidate genes. In seven recordings, individuals were already inside the nest box when video cameras began recording, thus we did not score sleep onset time. Similarly, in 20 recordings, individuals remained inside the nest box after video cameras stopped recording, and, as such, we did not score awakening time or morning latency to exit the box. Individuals without an identified sleep onset or awakening time were not assigned a relative sleep duration, midpoint of sleep, or proportion of time spent awake. Fourteen video recordings were of too low quality to score the proportion of time spent awake. Only sleep behaviors that were individually repeatable (credible intervals not including zero, with point estimates *r* > 0.05) in great tits were considered in this study: midpoint of sleep (*r* = 0.09), proportion of time spent awake during the night (*r* = 0.09), total sleep duration relative to night length (*r* = 0.06), morning awakening time (*r* = 0.08), and morning latency to exit the nest box (*r* = 0.66) (for behavioral definitions, see: Stuber *et al.* 2015, and File S1). Sample sizes for each behavior are given in Table 2.

### Identifying candidate genes

We performed a literature review to identify candidate genes of sleep from previous association studies in mammals and birds. We included candidate gene regions previously associated with behavioral or physiological sleep measures or circadian rhythms. In total, we identified 35 candidate genes from studies that demonstrated associations between genotypes and physiological or behavioral sleep phenotypes (see Table S1 for references). For nine of these candidate genes, we successfully developed microsatellite length polymorphisms (see *Microsatellite Identification*, below), and investigated their association with repeatable sleep traits in great tits. Variants in CLOCK and NPAS2 were included because they are core clock genes regulating circadian sleep-wake cycles in mammals and birds, and have been associated with timing of sleep onset, and offset, and sleep duration, and ADCYAP1 was investigated because of its influence on clock gene expression and nocturnal restlessness. SNPs in AANAT, a rate-limiting enzyme in melatonin production, which is regulated by the biological clock, have been associated with sleep onset time and duration in mammals, and awakening time and morning latency in birds. The CACNA1c gene was selected because of its association with sleep quality. Variants of the CREB1 gene may be related to the number of morning awakenings in men. We selected GRIA3 for its associations with both sleep duration, and number of awakenings in women. NPSR1’s endogenous ligand, neuropeptide S, is a promoter of wakefulness, and has been associated with sleep onset time. And, recently, a melanism-related gene, PCSK2, has been associated with rapid eye movement (REM) sleep in birds. Primer data for all candidate genes are provided in Table S2.

### Microsatellite identification

We queried the zebra finch (*Taeniopygia guttata*) assembly of the UCSC Genome Browser (http://www.genome.ucsc.edu/cgi-bin/hgGateway), searching for 35 candidate genes (Table S1). We examined the homologous regions of exons, introns, promoter regions, and regions 5000 bases upstream and downstream of candidate genes of the zebra finch for simple tandem repeat polymorphisms. Tandem repeat regions located in the zebra finch were compared with chicken (*Gallus gallus*), and medium ground finch (*Geospiza fortis*) sequences for cross species conservation. In the ADCYAP1, CLOCK, and NPAS2 candidate gene regions, we used microsatellites that were previously identified in blue tits (Steinmeyer *et al.* 2012).

We found usable tandem repeats for 17 of the candidate genes identified in Table S1. We designed forward and reverse primers for PCR amplification of tandem repeats (see File S1 for PCR details, and Table S2 for primer details) based on the zebra finch sequence and an aligned sequence from a second bird species (either chicken or medium ground finch) using PrimaClade (Gadberry *et al.* 2005). Primers were between 19 and 24 bases long, with one or two degenerate positions if necessary. We were able to design primers that functioned in great tits for 11 candidate genes. Once we amplified the target sequence of the great tit genome, we ran the PCR products of each candidate gene on a small sample (12–16 individuals) of presumably unrelated individuals on 1% agarose gel. If the bands on the gel displayed between-individual differences due to variance in length of the amplified products, we confirmed the presence of a polymorphism by running the fragments on a sequencer using fluorescently labeled primers. Two candidate genes did not show between-individual variation in microsatellite length (Table S1; Tandem repeat with no interindividual variation). We obtained the genotypes at all nine successfully identified candidate loci from 122 individual great tits for which sleep had been recorded (Table S1; Microsatellites used).

### Statistical analyses

For each microsatellite marker, we tested our sample including all individuals for deviations from Hardy-Weinberg equilibrium, and all pairs of microsatellites for linkage disequilibrium within years using Arlequin version 3 (Excoffier and Lischer 2010). We assessed the additive effect of the major allele of each microsatellite for association with each sleep parameter. This model assigns individual scores of 0, 1, or 2 based on the number of copies of the most abundant allele. This model has the potential to also capture an association of causal variants linked to the major allele. Second, for candidate genes that associated with sleep behavior using the additive major allele model, we modeled the mean allele length per individual, which assumes a linear effect of allele length and may suggest direct functionality of the microsatellite. Models were fit in the R programming environment version 2.14.1 (R Development Core Team 2011).

We estimated the associations between genotypes (encoding, see above) and sleep variables using linear mixed-effects models (package lme4; Gelman *et al.* 2015), with Gaussian error distribution and correcting for the effects of predictors known to have a strong influence on sleep behavior in our population (sex, month, and their interaction, and year: Stuber *et al.* 2015) partly due to seasonal changes in sleep behavior, and between-year environmental differences. We included plot, nest box nested within plot, individual identity, and recording date as random effects. The response variable morning latency was log-transformed to approximate normality. For the major allele copy number genotype encoding we fit five models (one for each of the sleep phenotypes), which included all candidate gene genotypes simultaneously as fixed effects. As a second step, we modeled the mean allele length genotype encoding of sleep behaviors where a significant major allele copy number effect was found. Mean genotype models included all noncandidate gene fixed effects as previously described, and only the candidate gene(s) significant in major allele copy number models. Using the sim function (package arm; Gelman *et al.* 2015), we simulated draws from the joint posterior distributions of the model parameters using noninformative priors. Based on 5000 simulations, we extracted the mean, and 95% credible intervals (CI) around the mean (Gelman and Hill 2007), which represent the parameter estimate and our uncertainty around this estimate. We assessed model fit by visual inspection of residual plots.

Furthermore, we tested the association of sleep behaviors, and nine random markers not expected to associate with sleep behaviors, to assess the number of associations that might be expected to arise by chance. We tested these markers [PmaTGAn33, PmaTGAn42, PmaTAGAn71, PmaTAGAn86, PmaD105, PmaD130 (Saladin *et al.* 2003); POCC6 (Bensch *et al.* 1997); Mcyμ4 (Double *et al.* 1997); Pca9 (Dawson *et al.* 2000)] using the same major allele copy number mixed-model structure but using the nine random markers instead of the nine candidate gene markers as fixed effects. Details regarding the random markers are presented in Araya-Ajoy (2015).

As population structure within the sample of individuals tested can confound associations (Balding 2006), we quantified genetic population substructure within both field seasons using the software program *Structure* (Falush *et al.* 2003; Pritchard *et al.* 2000) with default settings allowing for admixtured individuals and correlated allele frequencies between genetic clusters, and *Structure Harvester* (Earl and Vonholdt 2012) to combine the *Structure* output from 20 independent replications. The analysis is based on all 18 random and candidate markers, to test overall genetic structure with sufficient power.

### Data availability

Supplementary information contains genotyping processing and PCR condition information. Table S1 contains primer information, and Table S4 contains phenotype and genotype data used for analysis.

## Results

### Genetic polymorphisms

Microsatellite markers for candidate genes displayed between two and 13 alleles. All markers were in Hardy-Weinberg equilibrium except for NPSR1 in 2011/2012, and CREB1 in 2012/2013 (both not significant after Bonferroni correction, Table 1). After adjusting for multiple-testing, no pairs of microsatellites were in linkage-disequilibrium.

**Table 1:** Details regarding the microsatellite markers used in this study: polymorphism type, allele number, major allele frequency, observed (H_obs_) and expected (H_exp_) heterozygosity, and results of analyses of deviations from Hardy-Weinberg equilibrium (*P* values)

Candidate Gene	Polymorphism	No. of Alleles	Major Allele Frequency	H_obs_*^a^*	H_exp_*^a^*	*P**^a^*	H_obs_*^b^*	H_exp_*^b^*	*P**^b^*
AANAT	Trinucleotide—upstream	6	0.48	0.59	0.65	0.52	0.65	0.63	0.25
ADCYAP1	Dinucleotide—3′ UTR	4	0.53	0.62	0.66	0.94	0.65	0.63	0.43
CACNA1c	Trinucleotide—intron	7	0.51	0.69	0.64	0.24	0.67	0.64	0.26
CLOCK	Trinucleotide—exon	3	0.97	0.08	0.07	1.00	0.05	0.05	1.00
CREB1	Dinucleotide—intron/3′ UTR	5	0.96	0.09	0.09	1.00	0.07	0.09	0.02
GRIA3	Tetranucleotide—intron	3	0.93	0.15	0.14	1.00	0.15	0.15	1.00
NPAS2	Trinucleotide—exon	6	0.85	0.24	0.22	1.00	0.31	0.29	0.56
NPSR1	Pentanucleotide—upstream	13	0.19	0.88	0.87	0.03	0.89	0.87	0.42
PCSK2	Dinucleotide—intron	2	0.78	0.32	0.31	1.00	0.31	0.34	1.00

a *N* = 66 presumably unrelated individuals from 2011/2012.

b *N* = 61 presumably unrelated individuals from 2012/2013 (sample did not include any individuals from the previous season).

Random microsatellite markers displayed between three and 36 alleles. All markers were in Hardy-Weinberg equilibrium in both winter seasons. After adjusting for multiple-testing, no pair of microsatellite markers was in linkage-disequilibrium.

### Population substructure

Posterior probabilities of cluster analyses of all microsatellites assuming multiple genetic subclusters were not higher than models assuming no population substructure (K = 1) within seasons (see Figure S1). We conclude that there is no evidence for substructure in our population of great tits, and thus no detectable risk of confounding our genotype-phenotype associations.

### Genotype-phenotype associations

**Figure 1:**
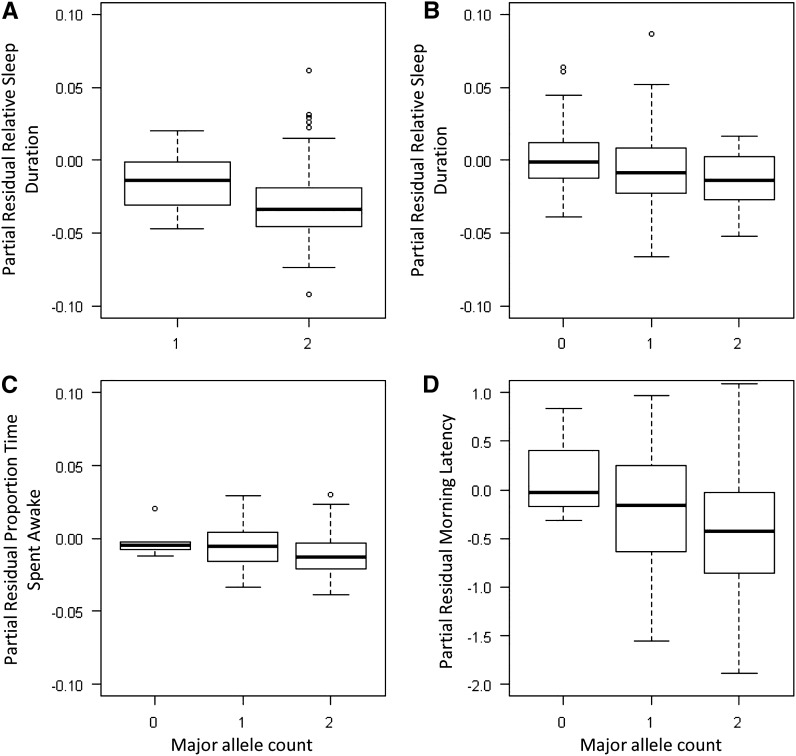
Effect of major allele copy number on sleep behavior after correcting for other fixed effects (see *Materials and Methods*). (A) CLOCK effect on sleep duration relative to night length; (B) NPSR1 effect on sleep duration relative to night length; (C) PCSK2 effect on proportion of time spent awake during the night; (D) NPAS2 effect on morning latency to exit the box (minutes; log-transformed). Shown are boxplots of the interquartile range of the data; the line inside each box represents the median effect and the whiskers extend to 1.5 × interquartile range.

We found support for significant negative associations between the major allele copy number in CLOCK and NPSR1, and sleep duration relative to night length (Table 2 and Figure 1, A and B), where each gene accounts for 6% of the between-individual variation, and 1% of the total phenotypic variation each. Major allele copy number in PCSK2 (Figure 1C) and NPAS2 (Figure 1D) were negatively associated with the proportion of time spent awake at night (accounting for 9% of the between-individual variation and 1% of the total phenotypic variation), and with morning latency to exit the nest box (accounting for 33% of the between-individual variation, and 2% of the total phenotypic variation), respectively. For comparison, two major allele associations between random markers (PmaTAGAn71, PmaD105) and sleep behaviors were detected; they were positively associated with morning latency and midpoint of sleep, respectively (Table S3).

**Table 2:** Parameter estimates from linear mixed-effects models of the additive effect of the major allele of microsatellites of nine candidate genes on variation in sleep behaviors

	Awakening Time*^a^*	Relative Sleep Duration*^b^*	Proportion Time Spent Awake*^c^*	Midpoint of Sleep*^d^*	Morning Latency*^e^*^,^*^f^*
Intercept	−7.53 (–24.27, 9.49)	1.06 (1.01, 1.10)	0.06 (0.02, 0.09)	−46.79 (–75.12, –16.32)	1.76 (0.33, 3.25)
AANAT	−1.55 (–3.52, 0.41)	0.001 (–0.006, 0.004)	0.002 (-0.002, 0.006)	−1.86 (–5.27, 1.36)	0.09 (–0.07, 0.25)
ADCYAP	0.64 (–1.25, 2.50)	−0.002 (–0.007, 0.003)	−0.009 (-0.005, 0.003)	1.95 (–1.57, 5.57)	−0.07 (–0.24, 0.09)
CACNA1C	1.12 (–0.60, 2.80)	0.001 (–0.003, 0.005)	−0.001 (-0.004, 0.003)	1.97 (–1.18, 5.02)	0.03 (–0.11, 0.17)
CLOCK	−3.30 (–8.25, 1.53)	**-0.016 (**–**0.03,** –**0.002)**	−0.001 (-0.013, 0.009)	5.87 (–3.68, 15.01)	−0.08 (–0.53, 0.36)
CREB1	−0.033 (–4.29, 3.76)	−0.002 (–0.01, 0.008)	0.001 (-0.007, 0.008)	−0.60 (–7.53, 6.71)	0.13 (–0.20, 0.47)
GRIA3	−1.49 (–5.08, 1.99)	−0.001 (–0.01, 0.008)	0.001 (-0.006, 0.009)	0.08 (–6.08, 6.59)	0.12 (–0.18, 0.41)
NPAS2	0.99 (–1.49, 3.43)	0.002 (–0.005, 0.009)	0.001 (-0.004, 0.006)	2.86 (–2.01, 7.54)	–**0.25 (**–**0.47,** –**0.04)**
NPSR1	−0.46 (–2.75, 1.89)	–**0.007 (**–**0.013,** –**0.0005)**	−0.002 (-0.007, 0.003)	0.43 (–3.68, 4.67)	0.07 (–0.13, 0.25)
PCSK2	−1.26 (–3.63, 1.11)	−0.004 (–0.01, 0.002)	–**0.005 (**–**0.01,** –**0.0006)**	1.38 (–2.69, 5.67)	−0.02 (–0.22, 0.18)
Sex (M)	–**3.89 (**–**6.15,** –**1.62)**	–**0.01 (**–**0.017,** –**0.004)**	−0.001 (–0.005, 0.004)	0.65 (–3.61, 5.04)	**-0.29 (**–**0.49,** –**0.09)**
Month	0.93 (–1.12, 2.92)	–**0.018 (**–**0.02,** –**0.01)**	−0.002 (–0.005, 0.001)	–**7.97 (**–**11.35,** –**4.57)**	−0.03 (–0.19, 0.13)
Year	**9.19 (4.90, 13.50)**	**0.04 (0.02, 0.05)**	–**0.02 (**–**0.03,** –**0.01)**	–**9.49 (**–**16.18,** –**4.57)**	−0.27 (–0.59, 0.03)
Sex (M) × Month	–**2.97 (**–**4.92,** –**1.06)**	–**0.01 (**–**0.015,** –**0.004)**	−0.002 (–0.006, 0.002)	1.04 (–2.80, 4.76)	0.007 (–0.17, 0.17)

Values are reported with 95% credible intervals. Significant effects are presented in bold.

a *N* = 221 observations; minutes relative to sunrise.

b *N* = 214 observations.

c *N* = 200 observations.

d *N* = 214 observations.

e *N* = 221 observations; minutes.

f log-transformed.

The mean allele length models revealed strong support (95% credible intervals did not overlap zero) for associations between CLOCK and PCSK2, and two sleep parameters (Table 3). After controlling for the effects of sex, month, and their interaction, year, and NPSR1 genotype, CLOCK microsatellite length negatively associated with relative sleep duration (Table 3). CLOCK explains 6% of the between-individual variation in relative sleep duration, and 1% of the total phenotypic variation. Because PCSK2 only has two allele variants in our population, the mean allele length model has the same encoding as the major allele copy number model. Thus, microsatellite length in PCSK2 also negatively associated with proportion of time spent awake at night, after correcting for fixed effects (compare Table 3 and Table 2). The mean genotypes of NPAS2 and NPSR1 were not associated with morning latency to exit the box, or relative sleep duration, respectively.

**Table 3:** Parameter estimates from linear mixed-effects models of the effects of mean microsatellite allele length of candidate genes significant from major allele analysis on variation in sleep behaviors

	Relative Sleep Duration*a*	Proportion Time Spent Awake*b*	Morning Latency*^c^*^,^*^d^*
Intercept	2.37 (1.09, 3.63)	1.00 (0.14, 1.83)	2.74 (–15.59, 20.97)
CLOCK	–**0.005 (**–**0.010,** –**0.0004)**	–	–
NPAS2	–	–	−0.01 (–0.11, 0.10)
NPSR1	0.001 (–0.0003, 0.0005)	–	–
PCSK2	–	–**0.005 (**–**0.009,** –**0.0005)**	–
Sex	–**0.01 (**–**0.02,** –**0.004)**	−0.0003 (–0.005, 0.004)	–**0.24 (**–**0.43,** –**0.04)**
Month	–**0.02 (**–**0.02,** –**0.01)**	−0.003 (–0.006, 0.0003)	−0.02 (–0.17, 0.12)
Year	**0.04 (0.02, 0.05)**	–**0.02 (**–**0.027,** –**0.15)**	−0.16 (–0.45, 0.12)
Sex × Month	–**0.009 (**–**0.014,** –**0.004)**	−0.001 (–0.005, 0.003)	0.03 (–0.14, 0.19)

Values are reported with 95% credible intervals. Significant effects are presented in bold.

a *N* = 214 observations.

b *N* = 200 observations.

c *N* = 214 observations; minutes.

d log-transformed.

## Discussion

We studied sleep behaviors in free-living great tits, and found evidence that repeatable components of sleep are associated with candidate genes for the biological clock and genes previously associated with sleep. Our literature search uncovered 35 candidate genes for sleep behaviors or biological timing. Half of these candidate genes did not have tandem repeats within the gene regions of interest and were not considered further. Candidate genes were largely selected based on studies of mammals, where microsatellites are more common (Primmer *et al.* 1997). Of the 17 remaining candidate genes with tandem repeats, we were unable to design working primers for six, possibly due to low sequence homology between the great tit and other avian species that we used to develop primers. Tandem repeats in two of the remaining candidate genes did not vary in length in our sample of individual great tits. The final set of nine candidate genes with microsatellites in the gene regions of interest had previously been associated with circadian timing systems, sleep timing, sleep duration, sleep quality, and physiological sleep. We detected four associations between candidate genes and the sleep behaviors relative sleep duration, proportion of time spent awake, and morning latency with the additive allele effect models, which may suggest a link between the major allele and another causal variant. Two of these associations were also replicated when using mean microsatellite allele length models; however, because PCSK2 has two alleles, these two genotype encoding models were necessarily the same.

The CLOCK poly-Q polymorphism has been identified in many passerine species (Caprioli *et al.* 2012; Johnsen *et al.* 2007; Liedvogel *et al.* 2012; Liedvogel and Sheldon 2010; Mueller *et al.* 2011), and has been associated with various phenotypes related to biological timing, including migration (Saino *et al.* 2015), and reproduction (Caprioli *et al.* 2012). CLOCK and NPAS2 appear to have partially redundant functions in the avian molecular clock (Cassone and Westneat 2012), but only a few studies have assessed the effects of both CLOCK and NPAS2 simultaneously (Mueller *et al.* 2011; Steinmeyer *et al.* 2012; this study). As in blue tits (Steinmeyer *et al.* 2012), we report three CLOCK alleles in the great tit. However, heterozygosity is much lower in our sample of individuals (0.05–0.08) than in the sample of blue tits (0.60; Steinmeyer *et al.* 2012), but similar to the mean observed heterozygosity in a different population of great tits (Liedvogel and Sheldon 2010), and in populations of barn swallows (*Hirundo rustica*) (Dor *et al.* 2011). Also in contrast with the blue tit study, we detect associations between the CLOCK polymorphism (located in a coding region) and circadian timing of sleep in great tits; individuals with increased major allele copy number, or longer microsatellite length had shorter sleep durations (relative to the length of the night). This finding agrees with previous work in mammals, relating CLOCK variants to both sleep onset (a component of sleep duration) and sleep duration (see references in Table S1). We did not detect any additional associations of CLOCK with other sleep behaviors assayed. We did detect an additive effect of the major NPAS2 allele on morning latency to exit the nest box, which may relate to sleep need or sleep inertia (Ferrara and De Gennaro 2000). Although previous work has highlighted the role of clock genes in sleep homeostasis (Naylor *et al.* 2000; Franken *et al.* 2001; Wisor *et al.* 2002) and sleep phase disorders (Xu *et al.* 2005; Toh *et al.* 2001), it is unclear why a clock gene should associate with this particular sleep phenotype (morning latency to exit the nest box). The circadian (regulating sleep timing) and homeostatic (tracking sleep need) regulatory systems of sleep do interact to generate overt sleep behaviors (Pace-Schott and Hobson 2002). Interpretation of this relationship would benefit from future work aimed at clarifying the relationship between morning latency as it is measured here, in free-living organisms, and sleep need or sleep inertia as measured in typical mammalian studies.

All other tested microsatellites were noncoding (upstream, intronic, or in untranslated exonic regions, Table 1), but their variation may still have functional consequences, for example, on expression dynamics through regulatory binding sites, mRNA degradation, or DNA methylation (Pieretti *et al.* 1991; Wang *et al.* 1994; Imagawa *et al.* 1995). The polymorphism could also be in linkage disequilibrium with a different functional polymorphism in the gene region influencing peptide structure or transcription level. Variation in major allele copy number in PCSK2 was associated with proportion of time spent awake at night. PCSK2 is responsible for α-melanocyte-stimulating hormone synthesis (Yoshihara *et al.* 2011) in the melanocortin system, and involved in skin pigmentation (Mundy 2005). Previous work in barn owls related the expression of this gene to variation in the amount of REM sleep during development (Scriba *et al.* 2013). Genetic variation leading to variation in hormone or neurotransmitter levels related to melanism may affect phenotypes by influencing developmental processes in the brain; juvenile owls with greater PCSK2 gene expression displayed reduced amounts of REM sleep, a more ‘precocial’ phenotype (Scriba *et al.* 2013). The genetic association between PCSK2 and sleep behavior supports previous evidence regarding a physiological measure of sleep and gene expression level, and gives weight to the credibility of such an unanticipated relationship.

We also found an additive effect of the NPSR1 major allele on relative sleep duration in the wild. This generally agrees with studies that show an effect of NPSR1 on sleep onset (a component of sleep duration; reference in Table S1), and on sleep duration in the elderly (Spada *et al.* 2014). Neuropeptide S (NPS) administration can elicit arousal (Xu *et al.* 2004), and modulate the expression of fear (Meis *et al.* 2008). The NPS receptor NPSR1 has been implicated in the regulation of the circadian system via knockout studies in mice which have revealed subsequent activity deficits (Duangdao *et al.* 2009), and NPS may regulate mRNA expression of other clock components (Acevedo *et al.* 2013). Further work is necessary to elucidate the implications of natural variation in NPS and its receptor’s function.

We did not detect a relationship between ADCYAP1 or AANAT and any sleep behaviors, although ADCYAP1 is purported to play a role in the biological clock (*e.g.*, clock gene expression: Nagy and Csernus 2007; circannual migratory behavior: Mueller *et al.* 2011; and reviewed in: Vaudry *et al.* 2009), and AANAT is a clock-controlled gene and rate-limiting enzyme in the production of melatonin (Kang *et al.* 2007). Only one study in birds has examined the relationship between ADCYAP1 and sleep behavior, and also found no relationship (Steinmeyer *et al.* 2012). But, the same study demonstrated marginal significance between two AANAT SNPs and awakening time, and longest sleep bout duration (Steinmeyer *et al.* 2012). The two exonic SNPs, however, are not directly comparable to our AANAT microsatellite located upstream of the gene.

Indeed, we were unable to test the same “significant” SNPs of this previous study, because SNPs are mostly species-specific, whereas our selected microsatellites are conserved across species. We selected microsatellites as representative markers within the candidate gene regions where the tested major allele has the potential to be linked to adjacent structural or regulatory variants of the gene. Further, the way we have identified the microsatellites makes it likely that they are functional themselves. All the short tandem repeat loci successfully tested were identified in the zebra finch genome, and appeared to be polymorphic in the great tit genome. Short tandem repeats of the nine loci could also be detected in the homologous regions of one to four other bird species among the five available avian genomes of the UCSC browser (*Gallus gallus*, *Meleagris gallopavo*, *Melopsittacus undulatus*, *Geospiza fortis*, *Taeniopygia guttata*; http://genome.ucsc.edu). Furthermore, four of these microsatellite loci (ADCYAP1, CLOCK, CREB1, NPAS2) are polymorphic in 33, 28, 23, and 20 of 37 tested bird species, respectively (unpublished data; Mueller *et al.* 2011). Such strongly conserved microsatellites in gene regions have often been associated with regulatory functions for gene expression (Sawaya *et al.* 2013; Abe and Gemmell 2014).

Although our approach with one representative marker per gene may be considered an incomplete candidate gene approach, a “complete” approach testing hundreds of SNPs within the gene region is plagued by the multiple testing problem similar to GWAS. Alternatively, testing only few nonsynonymous SNPs is criticized, because the majority of polymorphisms associated with complex traits are regulatory (Lowe and Reddy 2015). Selecting the local conserved (and thus presumably functional) microsatellite provides a strong foundation for association studies, has been successful in several instances (*e.g.*, Hammock and Young 2004; Mueller *et al.* 2013), and has the potential to complement GWAS where microsatellites are rarely tested (Press *et al.* 2014).

We were able to detect the effect of genes accounting for 1–2% of the observed variation in sleep behavior, and 6–33% of between-individual variation, which is typical for markers from candidate gene studies (Juhasz *et al.* 2009; Zhou *et al.* 2008; Tielbeek *et al.* 2012; Comings *et al.* 2000; Korsten *et al.* 2010; Mueller *et al.* 2014). Finally, we did not detect an association between CACNA1c, CREB1, or GRIA3, and sleep behaviors. This is perhaps less surprising, as previous work regarding the effect of these genes on sleep behavior were questionnaire-based human studies (Parsons *et al.* 2013), often in the context of disturbed sleep patterns (Utge *et al.* 2010, 2011).

Previous work in this population of great tits revealed primarily within-individual phenotypic correlations between some behavioral measures of sleep, indicating that these behaviors are not completely independent (Stuber *et al.* 2015). Of the sleep behaviors considered here, we found the strongest correlation between relative sleep duration and awakening time (*r* = 0.56; Stuber *et al.* 2015). Indeed, in both genotype encoding models where CLOCK is negatively associated with relative sleep duration, CLOCK also tends to negatively influence awakening time (credible intervals are nonsymmetric around zero, toward negative values), and may represent a between-individual correlation owing to these genetic effects. Additionally, midpoint of sleep was negatively associated with relative sleep duration (*r* = –0.40; Stuber *et al.* 2015). Again, our estimates of the effect of CLOCK on midpoint of sleep tend to be positive (credible intervals are nonsymmetric around zero, toward positive values) in both genotype encoding models. The proportion of time an individual spent awake at night was independent of other sleep behaviors (Stuber *et al.* 2015). Weak correlations between behavioral variables suggests that genes regulate specific aspects of sleep rather than having pleiotropic effects on all sleep behaviors, further suggesting that many of these sleep behaviors can evolve independently.

Cryptic population substructure in the test sample of both genetic, and phenotypic variation, can lead to pseudo-associations between genotypes and trait variation. It is a well-known confounding factor in association studies, and several methods have been developed to account for this. For example, “good practice” candidate gene studies should have a set of random markers to check test inflation, genetic substructure, or the ratio of expected chance results. In GWAS, all available markers are used for this purpose. Additionally, candidate gene studies should aim to be transparent in predefining and presenting the full list of genes and phenotypes tested for associations. The latter holds true for GWAS as well.

The candidate gene approach is well-suited to infer relationships between genes and conditions when effect sizes are small, allele frequencies are low, or the population sample is relatively small (Jorgensen *et al.* 2009). Moreover, some association details such as the effect of repeated trait measurements on the effect size can be easily investigated in candidate gene studies (Mueller *et al.* 2014). A candidate gene approach may provide insight to the generality of associations across vertebrates (Fidler *et al.* 2007; Shimada *et al.* 2004; Boehmler *et al.* 2007), the replicability of specific association sites in a gene within the same population (Korsten *et al.* 2010), and also the extent of heterogeneity across populations. GWAS are not designed to gain these insights, but, in contrast to candidate gene studies, GWAS are able to identify novel pathways not previously suspected in the etiology of a trait.

Contrariwise, GWAS, beyond being in a different cost category, do not serve as a general remedy for trait mapping. Although they may better account for cryptic genetic substructure, they embody a massive multiple testing problem (*i.e.*, millions of tests are performed when trait mapping is the aim). Although having One to two replication samples helps in this regard, such data sets can be accumulated only in long-term studies. The efficiency of such long-term projects in free-living animals, particularly detailing sleep behavior, is certainly reduced compared with typical studies conducted in humans or laboratory rodents. In this case, it is a preferred strategy to start with an information-based (hypothesis-driven) method such as the candidate gene approach that takes advantage of increased statistical power and biological understanding (Tabor *et al.* 2002) by testing the replication of known or potential “sleep” genes represented by an in-gene or nearby conserved microsatellite marker.

Our results add to the only other genotype-phenotype association study for sleep characteristics in birds (Steinmeyer *et al.* 2012). Further investigation of the genetic underpinning of sleep in birds is interesting because birds have independently evolved sleep states similar to those in mammals, providing a unique platform to help identify shared traits related to the function of sleep.

## Supplementary Material

Supporting Information
