# Supplementary material for: Genetic Correlates of Individual Differences in Sleep Behavior of Free-Living Great Tits (Parus major)
Source: G3 (Bethesda). 2016 Jan 5;6(3):599–607. doi: 10.1534/g3.115.024216 (PMC4777123; doi:10.1534/g3.115.024216)
Supplement: Supporting Information [file supp_6_3_599__index.html]

Genetic Correlates of Individual Differences in Sleep Behavior of Free-Living Great Tits (Parus major) — Supporting Information 

# Genetic Correlates of Individual Differences in Sleep Behavior of Free-Living Great Tits (*Parus major*)

## Supporting Information for Stuber *et al.*, 2016

**Files in this Data Supplement:**

- File S1 - Supplementary material. (.docx, 21 KB)
- Figure S1 - Mean posterior probabilities (+/- SD) of genotype data from 18 microsatellite markers given K subpopulations for a great tit population sampled during two field seasons. (.pptx, 68 KB)
- Table S1 - Genes that have been associated with sleep phenotypes or components of the circadian clock. (.docx, 83 KB)
- Table S2 - Primer information. (.docx, 14 KB)
- Table S3 - Parameter estimates from linear mixed-effects models of the additive effect of the major allele of nine random microsatellites on variation in sleep behaviors. (.docx, 15 KB)
- Table S4 - Genotype and phenotype information used for analysis. (.csv, 55 KB)
