# Supplementary material for: Genetic Correlates of Individual Differences in Sleep Behavior of Free-Living Great Tits (Parus major)
Source: G3 (Bethesda). 2016 Jan 5;6(3):599–607. doi: 10.1534/g3.115.024216 (PMC4777123; doi:10.1534/g3.115.024216)
Supplement: Supporting Information [file supp_g3.115.024216_FileS1.docx]

*Behavioral definitions*

Based on video recordings, we quantified individuals’ entry time relative to sunset, sleep onset relative to sunset, awakening time relative to sunrise and exit time relative to sunrise. Reference sunset and sunrise times were taken from Andechs, Germany.

We considered a bird asleep when it assumed the classical sleep posture with its beak tucked under the scapular feathers and its feathers fluffed up (Amlaner and Ball, 1983). We considered the bird awake if it was actively moving about, or its beak was out and facing forwards. Following Steinmeyer et al. (2010) and Stuber et al. (2014) we defined sleep onset as the time of the first sleep bout of at least 30 s (minutes relative to sunset), and awakening time as the end time of the final sleep bout of at least 30 s (minutes relative to sunrise). Evening latency to sleep was defined as the amount of time (min) between entering the nest box and falling asleep and morning latency to exit was defined as the amount of time (min) between awakening and exiting the nest box in the morning. Subsequently, we defined sleep duration relative to the night length as the amount of time between sleep onset and awakening time divided by the night length (amount of time between sunset and sunrise), and we calculated the relative midpoint of sleep as sleep onset time (relative to sunset) plus awakening time (relative to sunrise) (i.e. middle of sleep relative to night length; negative values represent earlier midpoints, while positive values represent later midpoints). We chose to define these variables relative to sunset, sunrise, or night length because the photoperiod changed dramatically over the study period.

To quantify the amount of time individuals spend awake during the night, we used a custom motion detection software program based on the AForgeVision [image processing](http://topics.sciencedirect.com/topics/page/Image_processing) library (aforgenet.com; Surhone et al., 2010) which we further developed at the Max Planck Institute for Ornithology to detect awakening bouts throughout the course of the night (for details see Stuber et al., 2014). We calculated the proportion of time spent awake during the night by summing the total durations of these awakening bouts and dividing by the individuals’ sleep.

*DNA sampling and extraction*

Each winter (2011 and 2012), we collected a small blood sample (~5µL) from the brachial vein of all adults roosting in our nest boxes. Blood samples were stored in Queen’s Lysis buffer (0.01 M Tris-HCl, 0.01 M NaCl, 0.01 M Na-EDTA, 1% n-Lauroylsarcosine, pH 8.0: Seutin et al., 1991) and genomic DNA was extracted using the NucleoSpin Blood Quick Pure Kit (Macherey-Nagel GmbH, Düren, Germany). Data were collected following permits obtained from the Bavarian regional government (Regierung von Oberbayern permit nos. 55.2-1-54-2532-140-11; 55.2-54-2531.2-7-2007).

*Genotyping*

Microsatellite amplifications were performed in multiplexed PCRs using the Qiagen Type-it Microsatellite PCR Kit (Qiagen, Hilden, Germany) and primer mixes containing five or six primer pairs. We used the program PrimaClade (www.umsl.edu/services/kellogg/primaclade.html), which is well-suited for designing primers for amplification from multi-species alignments. The forward or reverse primer of each pair was fluorescently labeled with 6-FAM, VIC, PET, or NED (Dye Set G5; Applied Biosystems, Darmstadt, Germany). Differences in amplification efficiency and dye strength of the primers were accommodated by adapting the primer concentrations in these mixes. Each 10μl multiplex PCR contained 20-80ng DNA, 5μl of 2x Type-it Microsatellite PCR Master Mix, and 1μl of primer mix. PCR cycling conditions began with 15 min initial denaturation at 95 ˚C, 28 cycles of 30s denaturation at 94 ˚C, 90s annealing at 57 or 56 ˚C, a 1 min extension at 72 ˚C, followed by a 30 min final extension at 60 ˚C. After amplification, 1.5μl of the PCR products were added to 13μl formamide containing the GeneScan 500 LIZ Size Standard, heat denatured, and run in a POP4 polymer in an ABI 3100 Genetic Analyzer (Applied Biosystems, Darmstadt, Germany) and alleles were assigned using the GeneMapper 4.0 software. AANAT primers did not function within a primer mix and had to be run separately following a different PCR protocol. PCR was conducted in a final volume of 10μl containing 1ul genomic DNA and 0.5μl Taq DNA polymerase (Fermentas) and a final concentration of 100μM dNTPs, 0.5μM of each of the forward and reverse primers and 1x Taq buffer with (NH_4_)_2_SO_4_ and 2.0mM MgCl_2_. Cycling conditions were: initial denaturation at 95 ˚C for 5 min, proceeding with 28 cycles of 95 ˚C for 30 s each, 30 s annealing at 57 ˚C, 1 min at 72 ˚C, followed by a final extension of 72 ˚C for 15min. PCR products for AANAT were then analyzed as previously described.

**References**

Amlaner CJ, and Ball NJ (1983) A Synthesis of Sleep in Wild Birds. Behaviour 87:85-119.

Seutin G, White BN, and Boag PT (1991) Preservation of Avian Blood and Tissue Samples for DNA Analyses. Can J Zool 69:82-90.

Steinmeyer C, Schielzeth H, Mueller JC, and Kempenaers B (2010) Variation in sleep behaviour in free-living blue tits, Cyanistes caeruleus: effects of sex, age and environment. Animal Behaviour 80:853-864.

Stuber EF, Grobis MM, Abbey-Lee R, Kempenaers B, Mueller JC, and Dingemanse NJ (2014) Perceived predation risk affects sleep behaviour in free-living great tits, Parus major. Animal Behaviour 98:157-165.

Surhone LM, Tennoe MT, and Henssonow SF (2010) AForge.NET: Artificial Intelligence, Computer Vision, .NET Framework. Betascript Publishing, Beau Bassin.
