# Supplementary material for: Genetic Correlates of Individual Differences in Sleep Behavior of Free-Living Great Tits (Parus major)
Source: G3 (Bethesda). 2016 Jan 5;6(3):599–607. doi: 10.1534/g3.115.024216 (PMC4777123; doi:10.1534/g3.115.024216)
Supplement: Supporting Information [file supp_g3.115.024216_FigureS1.pptx]

## Slide 1
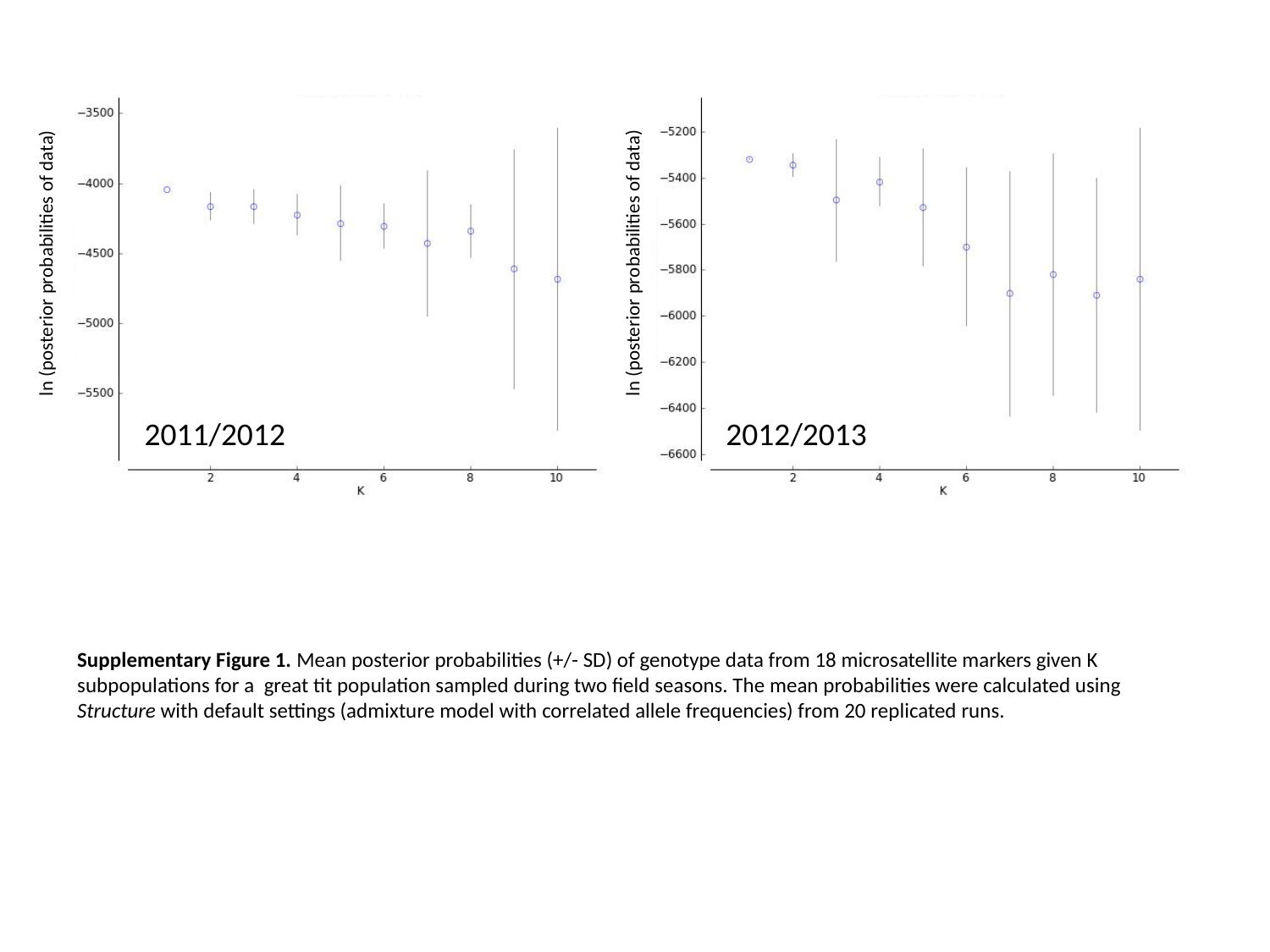

ln (posterior probabilities of data)
ln (posterior probabilities of data)
2011/2012
2012/2013
Supplementary Figure 1. Mean posterior probabilities (+/- SD) of genotype data from 18 microsatellite markers given K subpopulations for a great tit population sampled during two field seasons. The mean probabilities were calculated using Structure with default settings (admixture model with correlated allele frequencies) from 20 replicated runs.
