# Supplementary material for: Genetic Correlates of Individual Differences in Sleep Behavior of Free-Living Great Tits (Parus major)
Source: G3 (Bethesda). 2016 Jan 5;6(3):599–607. doi: 10.1534/g3.115.024216 (PMC4777123; doi:10.1534/g3.115.024216)
Supplement: Supporting Information [file supp_g3.115.024216_TableS1.docx]

**Table S1.** Genes that have been associated with sleep phenotypes or components of the circadian clock. References for work performed in birds are given in bold.

| **Candidate Genes** | **Gene name** | **Phenotype** | **References** |
| --- | --- | --- | --- |
| **Microsatellites used** |  |  |  |
| AANAT | Aralkylamine N-acetyltransferase | delayed sleep, sleep onset, awakening time, sleep duration, morning latency | ([Hohjoh et al. 2003](#_ENREF_13); [Wang et al. 2004](#_ENREF_34); [**Steinmeyer et al. 2012**](#_ENREF_29)) |
| ADCYAP1 | Adenylate cyclase activating polypeptide | clock timing, nocturnal restlessness | ([**Nagy and Csernus 2007**](#_ENREF_19); [**Mueller et al. 2011**](#_ENREF_18)**;** [**Steinmeyer et al. 2012**](#_ENREF_29)) |
| CACNA1c | L-type voltage-dependent calcium channel | sleep quality, latency to sleep | ([Parsons et al. 2013](#_ENREF_22); [Byrne et al. 2013](#_ENREF_5)) |
| CLOCK | Circadian Locomotor Output Cycles Kaput | sleep onset, duration | ([Gottlieb et al. 2007](#_ENREF_11); [Allebrandt et al. 2010](#_ENREF_2); [Evans et al. 2013](#_ENREF_9); [Kripke et al. 2010](#_ENREF_15); [**Steinmeyer et al. 2012**](#_ENREF_29)) |
| CREB1 | cAMP responsive element binding protein 1 | nighttime awakenings | ([Utge et al. 2010a](#_ENREF_32); [**Steinmeyer et al. 2012**](#_ENREF_29)) |
| GRIA3 | Glutamate receptor, ionotropic, AMPA 3 | nighttime awakenings, duration | ([Utge et al. 2011](#_ENREF_31); [Utge et al. 2010a](#_ENREF_32)) |
| NPAS2 | Neuronal PAS domain protein 2 | sleep onset, sleep offset | ([Evans et al. 2013](#_ENREF_9); [**Steinmeyer et al. 2012**](#_ENREF_29)) |
| NPSR1 | Neuropeptide S receptor 1 | sleep onset | ([Gottlieb et al. 2007](#_ENREF_11)) |
| PCSK2 | Proprotein convertase subtilisin, kexin type 2 | REM amount | ([**Scriba et al. 2013**](#_ENREF_28)) |
| **Tandem repeat with no inter-individual variation** |  |  |  |
| ABCC9 | ATP-binding cassette, sub-family C (CFTR, MRP), member 9 | duration | ([Allebrandt et al. 2013](#_ENREF_1)) |
| TEF | Thyrotroph embryonic factor | nighttime awakenings | ([Hua et al. 2012](#_ENREF_14)) |
| **Tandem repeat without working primers** |  |  |  |
| CK1d | Casein kinase 1 delta | familial advanced sleep phase syndrome; sleep onset, sleep offset | ([Xu et al. 2005](#_ENREF_35)) |
| GNB3 | Guanine nucleotide binding protein (G protein), beta polypeptide 3 | wake after sleep onset, sleep bout length, nighttime awakenings | ([Evans et al. 2013](#_ENREF_9)) |
| HOMER1a | Homer protein homolog 1 | slow-wave activity | ([Mackiewicz et al. 2008](#_ENREF_17)) |
| MAOA | Monoamine oxidase A | nighttime awakenings, latency to sleep, duration | ([Brummett et al. 2007](#_ENREF_4); [Craig et al. 2006](#_ENREF_7)) |
| PED4D | Phosphodieterase 4D | latency to sleep | ([Gottlieb et al. 2007](#_ENREF_11)) |
| PER2 | Period circadian clock 2 | sleep onset, sleep offset | ([Carpen et al. 2006](#_ENREF_6); [Steinmeyer et al. 2012](#_ENREF_29)) |
| **No tandem repeats** |  |  |  |
| ADA | Adenosine deaminase | nighttime awakenings | ([Retey et al. 2005](#_ENREF_24)) |
| ADORA2A | Adenosine A2a receptor | nighttime awakenings, TTA | ([Nova et al. 2012](#_ENREF_20)) |
| ARNTL | Aryl hydrocarbon receptor nuclear translocator-like | sleep onset, sleep offset | ([Evans et al. 2013](#_ENREF_9)) |
| CK1e | Casein kinase 1 epsilon | sleep onset, sleep offset, morning latency | ([Takano et al. 2004](#_ENREF_30); [Steinmeyer et al. 2012](#_ENREF_29)) |
| CRHR1 | Corticotropin releasing hormone receptor 1 | nighttime awakenings | ([Utge et al. 2010a](#_ENREF_32)) |
| CSNK2A2 | Casein kinase 2, alpha prime polypeptide | sleep onset | ([Gottlieb et al. 2007](#_ENREF_11)) |
| HCRT | Preprohypocretin | sudden sleep onset | ([Rissling et al. 2005](#_ENREF_25)) |
| HLA-DR1 | Human leucocyte antigen DR1 | delayed sleep phase syndrome; sleep onset, sleep offset | ([Hohjoh et al. 1999](#_ENREF_12)) |
| NT5E | 5'-ectonucleotidase | sleep offset, nighttime awakenings | ([Gass et al. 2010](#_ENREF_10)) |
| OPN4 | Melanopsin | sleep onset, sleep offset | ([Roecklein et al. 2009](#_ENREF_26); [Roecklein et al. 2012](#_ENREF_27)) |
| PER1 | Period circadian clock 1 | sleep onset, sleep offset | ([Carpen et al. 2006](#_ENREF_6)) |
| PER3 | Period circadian clock 3 | delayed sleep phase syndrome; sleep onset, sleep offset | ([Archer et al. 2003](#_ENREF_3); [Ebisawa et al. 2001](#_ENREF_8); [Lazar et al. 2012](#_ENREF_16); [Pereira et al. 2005](#_ENREF_23)) |
| PROK2 | Prokineticin 2 | duration | ([Gottlieb et al. 2007](#_ENREF_11)) |
| SLC28A1 | Solute carrier family 28, member 1, CNT1 | sleep offset, nighttime awakenings | ([Gass et al. 2010](#_ENREF_10)) |
| SLC29A1 | Solute carrier family 29, member 2, ENT2 | sleep offset, nighttime awakenings | ([Gass et al. 2010](#_ENREF_10)) |
| SLC29A3 | Solute carrier family 29, member 4, ENT4 | sleep offset, nighttime awakenings | ([Gass et al. 2010](#_ENREF_10)) |
| TIMELESS | Timeless circadian clock | sleep offset | ([Utge et al. 2010b](#_ENREF_33)) |
| TRIB1 | Tribbles homolog 1 | duration, slow wave sleep | ([Ollila et al. 2012](#_ENREF_21)) |

Allebrandt, K.V., N. Amin, B. Muller-Myhsok, T. Esko, M. Teder-Laving *et al.*, 2013 A K(ATP) channel gene effect on sleep duration: from genome-wide association studies to function in Drosophila. *Mol Psychiatry* 18 (1):122-132.

Allebrandt, K.V., M. Teder-Laving, M. Akyol, I. Pichler, B. Muller-Myhsok *et al.*, 2010 CLOCK Gene Variants Associate with Sleep Duration in Two Independent Populations. *Biological Psychiatry* 67 (11):1040-1047.

Archer, S.N., D.L. Robillard, D.J. Skene, M. Smits, A. Williams *et al.*, 2003 A length polymorphism in the circadian clock gene Per3 is linked to delayed sleep phase syndrome and extreme diurnal preference. *Sleep* 26:A109-A109.

Brummett, B.H., A.D. Krystal, I.C. Siegler, C. Kuhn, R.S. Surwit *et al.*, 2007 Associations of a regulatory polymorphism of monoamine oxidase-A gene promoter (MAOA-uVNTR) with symptoms of depression and sleep quality. *Psychosomatic Medicine* 69 (5):396-401.

Byrne, E.M., P.R. Gehrman, S.E. Medland, D.R. Nyholt, A.C. Heath *et al.*, 2013 A genome-wide association study of sleep habits and insomnia. *American Journal of Medical Genetics Part B-Neuropsychiatric Genetics* 162B (5):439-451.

Carpen, J.D., M. von Schantz, M. Smits, D.J. Skene, and S.N. Archer, 2006 A silent polymorphism in the PER1 gene associates with extreme diurnal preference in humans. *Journal of Human Genetics* 51 (12):1122-1125.

Craig, D., D.J. Hart, and A.P. Passmore, 2006 Genetically increased risk of sleep disruption in Alzheimer's disease. *Sleep* 29 (8):1003-1007.

Ebisawa, T., M. Uchiyama, N. Kajimura, K. Mishima, Y. Kamei *et al.*, 2001 Association of structural polymorphisms in the human period3 gene with delayed sleep phase syndrome. *Embo Reports* 2 (4):342-346.

Evans, D.S., N. Parimi, C.M. Nievergelt, T. Blackwell, S. Redline *et al.*, 2013 Common Genetic Variants in ARNTL and NPAS2 and at Chromosome 12p13 are Associated with Objectively Measured Sleep Traits in the Elderly. *Sleep* 36 (3):431-446.

Gass, N., H.M. Ollila, S. Utge, T. Partonen, E. Kronholm *et al.*, 2010 Contribution of adenosine related genes to the risk of depression with disturbed sleep. *Journal of Affective Disorders* 126 (1-2):134-139.

Gottlieb, D.J., G.T. O'Connor, and J.B. Wilk, 2007 Genome-wide association of sleep and circadian phenotypes. *Bmc Medical Genetics* 8.

Hohjoh, H., Y. Takahashi, Y. Hatta, H. Tanaka, T. Akaza *et al.*, 1999 Possible association of human leucocyte antigen DR1 with delayed sleep phase syndrome. *Psychiatry and Clinical Neurosciences* 53 (4):527-529.

Hohjoh, H., M. Takasu, K. Shishikura, Y. Takahashi, Y. Honda *et al.*, 2003 Significant association of the arylalkylamine N-acetyltransferase (AA-NAT) gene with delayed sleep phase syndrome. *Neurogenetics* 4 (3):151-153.

Hua, P., W.G. Liu, Y.Y. Zhao, H.X. Ding, L. Wang *et al.*, 2012 Tef polymorphism is associated with sleep disturbances in patients with Parkinson's disease. *Sleep Medicine* 13 (3):297-300.

Kripke, D.F., F.F. Shadan, A. Dawson, J.W. Cronin, S.M. Jamil *et al.*, 2010 Genotyping Sleep Disorders Patients. *Psychiatry Investigation* 7 (1):36-42.

Lazar, A.S., A. Slak, J.C.Y. Lo, N. Santhi, M. von Schantz *et al.*, 2012 Sleep, Diurnal Preference, Health, and Psychological Well-being: A Prospective Single-Allelic-Variation Study. *Chronobiology International* 29 (2):131-146.

Mackiewicz, M., B. Paigen, N. Naidoo, and A.I. Pack, 2008 Analysis of the QTL for sleep homeostasis in mice: Homer1a is a likely candidate. *Physiological Genomics* 33 (1):91-99.

Mueller, J.C., F. Pulido, and B. Kempenaers, 2011 Identification of a gene associated with avian migratory behaviour. *Proceedings of the Royal Society B-Biological Sciences* 278 (1719):2848-2856.

Nagy, A.D., and V.J. Csernus, 2007 The role of PACAP in the control of circadian expression of clock genes in the chicken pineal gland. *Peptides* 28 (9):1767-1774.

Nova, P., B. Hernandez, A.S. Ptolemy, and J.M. Zeitzer, 2012 Modeling caffeine concentrations with the Stanford Caffeine Questionnaire: Preliminary evidence for an interaction of chronotype with the effects of caffeine on sleep. *Sleep Medicine* 13 (4):362-367.

Ollila, H.M., S. Utge, E. Kronholm, V. Aho, W. Van Leeuwen *et al.*, 2012 TRIB1 constitutes a molecular link between regulation of sleep and lipid metabolism in humans. *Translational Psychiatry* 2.

Parsons, M.J., K.J. Lester, N.L. Barclay, P.M. Nolan, T.C. Eley *et al.*, 2013 Replication of Genome-Wide association studies (GWAS) loci for sleep in the British G1219 cohort. *American Journal of Medical Genetics Part B-Neuropsychiatric Genetics* 162B (5):431-438.

Pereira, D.S., S. Tufik, F.M. Louzada, A.A. Benedito-Silva, A.R. Lopez *et al.*, 2005 Association of the length polymorphism delayed sleep-phase syndrome: Does in the human Per3 gene with the latitude have an influence upon it? *Sleep* 28 (1):29-32.

Retey, J.V., M. Adam, E. Honegger, R. Khatami, U.F.O. Luhmann *et al.*, 2005 A functional genetic variation of adenosine deaminase affects the duration and intensity of deep sleep in humans. *Proceedings of the National Academy of Sciences of the United States of America* 102 (43):15676-15681.

Rissling, I., Y. Korner, F. Geller, K. Stiasny-Kolster, W.H. Oertel *et al.*, 2005 Preprohypocretin polymorphisms in Parkinson disease patients reporting "Sleep attacks". *Sleep* 28 (7):871-875.

Roecklein, K.A., K.J. Rohan, W.C. Duncan, M.D. Rollag, N.E. Rosenthal *et al.*, 2009 A missense variant (P10L) of the melanopsin (OPN4) gene in seasonal affective disorder. *Journal of Affective Disorders* 114 (1-3):279-285.

Roecklein, K.A., P.M. Wong, P.L. Franzen, B.P. Hasler, W.M. Wood-Vasey *et al.*, 2012 Melanopsin Gene Variations Interact With Season to Predict Sleep Onset and Chronotype. *Chronobiology International* 29 (8):1036-1047.

Scriba, M.F., A.L. Ducrest, I. Henry, A.L. Vyssotski, N.C. Rattenborg *et al.*, 2013 Linking melanism to brain development: expression of a melanism-related gene in barn owl feather follicles covaries with sleep ontogeny. *Frontiers in Zoology* 10.

Steinmeyer, C., B. Kempenaers, and J.C. Mueller, 2012 Testing for associations between candidate genes for circadian rhythms and individual variation in sleep behaviour in blue tits. *Genetica* 140 (4-6):219-228.

Takano, A., M. Uchiyama, N. Kajimura, K. Mishima, Y. Inoue *et al.*, 2004 Sequence variations in human casein kinase1epsilon gene and circadian rhythm sleep disorders. *American Journal of Medical Genetics Part B-Neuropsychiatric Genetics* 130B (1):152-153.

Utge, S., E. Kronholm, T. Partonen, P. Soronen, H.M. Ollila *et al.*, 2011 Shared Genetic Background for Regulation of Mood and Sleep: Association of GRIA3 with Sleep Duration in Healthy Finnish Women. *Sleep* 34 (10):1309-U1341.

Utge, S., P. Soronen, T. Partonen, A. Loukola, E. Kronholm *et al.*, 2010a A Population-Based Association Study of Candidate Genes for Depression and Sleep Disturbance. *American Journal of Medical Genetics Part B-Neuropsychiatric Genetics* 153B (2):468-476.

Utge, S.J., P. Soronen, A. Loukola, E. Kronholm, H.M. Ollila *et al.*, 2010b Systematic Analysis of Circadian Genes in a Population-Based Sample Reveals Association of TIMELESS with Depression and Sleep Disturbance. *Plos One* 5 (2).

Wang, G.Y., C.G.L. Lee, and E.J.D. Lee, 2004 Genetic variability of arylalkylamine-N-acetyl-transferase (AA-NAT) gene and human sleep/wake pattern. *Chronobiology International* 21 (2):229-237.

Xu, Y., Q.S. Padiath, R.E. Shapiro, C.R. Jones, S.C. Wu *et al.*, 2005 Functional consequences of a CKI delta mutation causing familial advanced sleep phase syndrome. *Nature* 434 (7033):640-644.
