# Supplementary material for: Genetic Correlates of Individual Differences in Sleep Behavior of Free-Living Great Tits (Parus major)
Source: G3 (Bethesda). 2016 Jan 5;6(3):599–607. doi: 10.1534/g3.115.024216 (PMC4777123; doi:10.1534/g3.115.024216)
Supplement: Supporting Information [file supp_g3.115.024216_TableS2.docx]

**Table S2.** Primer information.

| **Gene name** | **Forward Primer** | **Reverse Primer** | **T_m_ °C forward/reverse** | **Expected length bp** | **MgCl_2_ mM** | **T_a_ °C** |
| --- | --- | --- | --- | --- | --- | --- |
| **AANAT** | CACTGCATCCYTCCCACGA | GAGGAGCTCGGCTGGAATAAA | 59.9 / 59.8 | 400 | 2 | 57 |
| **ABCC9** | CCCAGTGAAATCCCTGAAGTTCTTT | GCCTGTCAAGCTTTYCCCTAGTTT | 61.3 /61.9 | 450 | 2 | 59 |
| **ADCYAP1^a^** | GATGTGAGTAACCAGCCACT | ATAACACAGGAGCGGTGA | 57/53 | 166 | 1.5 | 51 |
| **CACNA1C** | TCGCCAACCAATCCGAGCTCAG | AATGRAGAAACCACCCCYACCCC | 64 / 64.2 | 380 | 2 | 59 |
| **CLOCK^a^** | TTTTCTCAAGGTCAGCAGCTTGT | CTGTAGGAACTGTTGYGGKTGCTG | 58/64 | 285 | 1.5 | 58 |
| **CREB1^a^** | GGTCAGGCAGTTAAGATATTG | GTCTTACCAGTGGTTCCTTTAR | 55/57 | 556 | 2 | 53 |
| **GRIA3** | ACTGCTGGAAAGATGCCCCTTAG | GACAAGCTGCCTTTGTAAAYCCTCT | 62.4 / 62.2 | 250 | 2 | 60 |
| **NPAS2^a^** | CTGTGGTAAATTTGATGATTCTGA | ACACCAAGTTCTTTGCACAATG | 55/56 | 184 | 2 | 55 |
| **NPSR1** | TATCTCCCCATAGTGGCATGG | CAYCTCCACCTATTGCATTYCACA | 59.8 / 61.0 | 375 | 2 | 57 |
| **PCSK2** | TTTGCACTTTKCTGTCTCACCAG | GTTTTCTGCAAGTCACTGTTTTCC | 59.8 / 59.3 | 160 | 2 | 57 |
| **TEF** | GGGAGGCATKAATTTGACTCTCC | GAAAGCACAGCTGATGGGGAA | 61.5 / 59.8 | 390 | 2 | 58 |

^a^= primers published in Steinmeyer et al. 2009

Steinmeyer C, J.C. Mueller, and B. Kempenaers. 2009. Search for informative polymorphisms in candidate genes: clock genes and circadian behaviour in blue tits. Genetica 136:109–117
