# Supplementary material for: Genetic Correlates of Individual Differences in Sleep Behavior of Free-Living Great Tits (Parus major)
Source: G3 (Bethesda). 2016 Jan 5;6(3):599–607. doi: 10.1534/g3.115.024216 (PMC4777123; doi:10.1534/g3.115.024216)
Supplement: Supporting Information [file supp_g3.115.024216_TableS3.docx]

| **Table S3.** Parameter estimates from linear mixed-effects models of the additive effect of the major allele of nine random microsatellites on variation in sleep behaviors. Values are reported with 95% credible intervals. Significant effects are presented in bold. | | | | | |
| --- | --- | --- | --- | --- | --- |
|  | **Awake time^1^** | **Relative sleep duration^2^** | **Proportion time spent awake^3^** | **Midpoint of sleep^4^** | **Morning latency^5*^** |
| **Intercept** | -15.85 (-20.87, -10.23) | 1.02 (1.01, 1.04) | 0.05 (0.04, 0.06) | -28.33 (-37.59, -19.39) | 1.36 (0.95, 1.77) |
| **PmaTGAn42** | 0.09 (-1.83, 2.18) | 0.002 (-0.004, 0.008) | -0.001 (-0.005, 0.004) | -0.81 (-4.37, 2.74) | -0.08 (-0.25, 0.09) |
| **POCC6** | 0.47 (-1.74, 2.79) | 0.002 (-0.005, 0.008) | 0.002 (-0.002, 0.007) | -0.11 (-4.04, 4.03) | -0.13 (-0.32, 0.06) |
| **Mcyµ4** | -0.17 (-1.78, 1.43) | -0.001 (-0.006, 0.004) | 0.001 (-0.003, 0.005) | -0.21 (-3.17, 2.71) | 0.08 (-0.07, 0.23) |
| **PmaD130** | -1.68 (-3.49, 0.18) | -0.000 (-0.005, 0.005) | -0.001 (-0.005, 0.003) | -1.86 (-5.05, 1.40) | 0.12 (-0.03, 0.29) |
| **PmaTGA33** | 1.09 (-1.01, 3.25) | -0.001 (-0.007, 0.004) | -0.002 (-0.006, 0.003) | 1.83 (-2.28, 5.77) | -0.12 (-0.30, 0.06) |
| **PmaTAGAn86** | -1.57 ( -3.79, 0.61) | -0.005 (-0.01, 0.001) | 0.000 (-0.005, 0.005) | 1.86 (-2.20, 5.98) | -0.11 (-0.29, 0.08) |
| **PmaD105** | 0.41 (-1.68, 2.45) | -0.005 (-0.01, 0.001) | 0.003 (-0.002, 0.008) | **5.40 (1.42, 9.52)** | 0.03 (-0.16, 0.21) |
| **PmaTAGAn71** | -0.35 (-2.38, 1.61) | 0.001 (-0.005, 0.006) | 0.000 (-0.004, 0.005) | -1.78 (-5.64, 1.85) | **0.25 (0.09, 0.43)** |
| **Pca9** | 0.65 (-0.99, 2.32) | 0.001 (-0.004, 0.006) | -0.000 (-0.004, 0.004) | 1.12 (-1.96, 4.41) | 0.08 (-0.06, 0.23) |
| **Sex (M)** | **-4.33 (-6.70, -2.01)** | **-0.01 (-0.018, -0.004)** | -0.000 (-0.005, 0.005) | -0.32 (-4.65, 3.81) | -0.18 (-0.39, 0.02) |
| **Month** | 0.76 (-1.41, 2.76) | **-0.018 (-0.02, -0.01)** | -0.003 (-0.006, 0.001) | **-8.05 (-11.48, -4.70)** | -0.04 (-0.19, 0.10) |
| **Year** | **8.82 (4.50, 13.16)** | **0.04 (0.02, 0.05)** | **-0.02 (-0.03, -0.01)** | **-10.25 (-16.96, -3.37)** | -0.16 (-0.46, 0.12) |
| **Sex (M) × Month** | **-2.81 (-4.78, -0.84)** | **-0.01 (-0.015, -0.004)** | -0.001 (-0.005, 0.003) | 0.80 (-2.93, 4.47) | 0.02 (-0.14, 0.18) |

1: N=221 observations; minutes relative to sunrise

2: N=214 observations

3: N=200 observations

4: N=214 observations

5: N=221 observations; minutes

*:log-tansformed
